# Mechanisms of Rhodopsin-Related Inherited Retinal Degeneration and Pharmacological Treatment Strategies

**DOI:** 10.3390/cells14010049

**Published:** 2025-01-04

**Authors:** Maria Azam, Beata Jastrzebska

**Affiliations:** 1 Department of Pharmacology, School of Medicine, Case Western Reserve University, 10900 Euclid Ave., Cleveland, OH 44106, USA; 2 Cleveland Center for Membrane and Structural Biology, Case Western Reserve University, 10900 Euclid Ave., Cleveland, OH 44106, USA

**Keywords:** misfolding, oxidative stress, neuroinflammation, photoreceptor, retinal degeneration, rhodopsin

## Abstract

Retinitis pigmentosa (RP) is a hereditary disease characterized by progressive vision loss ultimately leading to blindness. This condition is initiated by mutations in genes expressed in retinal cells, resulting in the degeneration of rod photoreceptors, which is subsequently followed by the loss of cone photoreceptors. Mutations in various genes expressed in the retina are associated with RP. Among them, mutations in the rhodopsin gene (*RHO*) are the most common cause of this condition. Due to the involvement of numerous genes and multiple mutations in a single gene, RP is a highly heterogeneous disease making the development of effective treatments particularly challenging. The progression of this disease involves complex cellular responses to restore cellular homeostasis, including the unfolded protein response (UPR) signaling, autophagy, and various cell death pathways. These mechanisms, however, often fail to prevent photoreceptor cell degradation and instead contribute to cell death under certain conditions. Current research focuses on the pharmacological modulation of the components of these pathways and the direct stabilization of mutated receptors as potential treatment strategies. Despite these efforts, the intricate interplay between these mechanisms and the diverse causative mutations involved has hindered the development of effective treatments. Advancing our understanding of the interactions between photoreceptor cell death mechanisms and the specific genetic mutations driving RP is critical to accelerate the discovery and development of therapeutic strategies for this currently incurable disease.

## 1. Introduction

### 1.1. Background

The retina is a multilayered tissue located at the back of the eye, crucial for vision by processing light photons into three-dimensional images. It is connected to the brain via the optic nerve and forms part of the central nervous system (CNS) [1]. The retina is the most metabolically demanding tissue in the human body, consuming more oxygen than any other tissue, and thus it is highly susceptible to oxidative stress [2]. Maintaining retinal homeostasis requires a precisely regulated supply of oxygen and nutrients, as well as the timely removal of toxic metabolites to minimize oxidative damage. Dual blood circulation, comprising the choroid and branches of the ophthalmic artery, adjacent to the retina, meets these demanding requirements [2].

The retina consists of ten distinct layers and contains three main types of cells, namely photoreceptors, neuronal cells, and glial cells, all of which play essential roles in vision [1]. Among the photoreceptors, rods and cones are located in the outer retina. The more abundant rod cells are sensitive to dim light, while cones, concentrated in the macula (the central part of the retina), are sensitive to bright light and are responsible for color discrimination. Cones are further divided into three subtypes based on their sensitivity to specific wavelengths of light: long-wavelength cones detecting red light, middle-wavelength cones detecting green light, and short-wavelength cones detecting blue light.

Neuronal cells in the retina include ganglion cells, bipolar neurons, amacrine cells, and horizontal cells, all of which are involved in the processing of visual signals. Glial cells, such as microglia and Müller glia, serve as the retina’s resident immune cells, continuously monitoring and supporting retinal cells to maintain a healthy environment [3,4].

**Figure 1:**
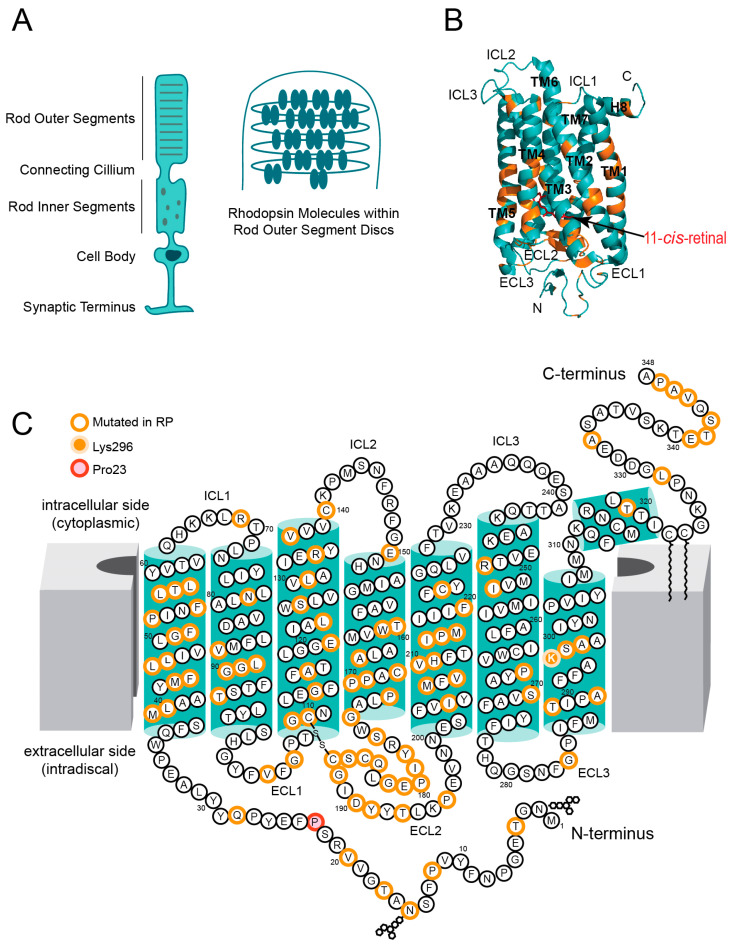
Schematic rod photoreceptor and rhodopsin structure. (**A**) The schematic representation of the rod photoreceptor cell (left panel) and a close-up of rod outer segment disc membranes with rhodopsin (Rho) molecules. (**B**) The structure of bovine Rho. The PDB ID:1GZM was used to show the side view of bovine Rho in the dark state. Transmembrane helices are labeled TM1–7. Cytoplasmic helix 8 is labeled H8. Extracellular (intradiscal) loops connecting TM helices on the ligand-binding site of the receptor are labeled ECL1, ECL2, and ECL3. Intracellular (cytoplasmic) loops, connecting TM helices on the effector binding site of the receptor are labeled ICL1, ICL2, and ICL3. 11-*cis*-retinal is shown as red sticks. The location of residues mutated in retinitis pigmentosa (RP) is shown in orange. (**C**) Two-dimensional representation of human Rho structure. Residues mutated in RP are indicated with orange circles. The Lys296, which covalently binds the 11-*cis*-retinal, is shown with a yellow circle filled with orange. The P23H mutation is shown with a red circle filled with orange.

Photoreceptors are highly specialized neurons with distinct inner and outer segments (Figure 1A) [5]. The inner segments are responsible for protein synthesis, while the outer segments contain numerous parallel discs that house the critical components for phototransduction, including visual pigments such as rhodopsin (Rho) in rods and cone opsins in cones. These photoreceptors are closely associated with retinal pigment epithelial (RPE) cells, which are indispensable for retinal homeostasis. RPE cells perform several key functions, including the renewal of photoreceptor outer segments, the regeneration of 11-*cis*-retinal (the chromophore essential for Rho and cone opsins), detoxification of the retina, and the transport of nutrients and oxygen from blood circulation to photoreceptors [6].

Dysfunction of key proteins in either photoreceptors or RPE cells can result in various retinal pathologies, highlighting the importance of their interdependence for maintaining visual health. This review discusses recent progress in understanding the underlying mechanisms of photoreceptor cell death in RP and summarizes pharmacological treatment strategies predominantly related to mutations in *RHO*.

### 1.2. Inherited Retinal Degenerations

Inherited retina degeneration is a clinically and genetically heterogeneous group of blinding diseases caused by mutations in genes encoding proteins critical for retinal development, structure, and function [7,8,9]. The most common, progressive form of hereditary retinal dystrophy is retinitis pigmentosa (RP), which can be inherited as autosomal dominant RP (adRP) (15–25%), autosomal recessive RP (arRP) (5–20%), or X-linked RP (5–15%) [10]. About 40–50% of RP cases have still unknown patterns. RP affects nearly 1 in 4000 individuals in the US and over 2 million around the globe. Mutations in proteins involved in phototransduction, folding, cell trafficking, and Rho’s retinal chromophore recycling pathways may be involved in the pathophysiology of RP “https://retnet.org/ (accessed on 30 September 2024)”. During the initial phase of RP, more peripherally localized rod photoreceptors become distorted, resulting in “so-called” tunnel vision and night blindness. However, in the late phase, cone photoreceptors also deteriorate, which eventually leads to complete blindness.

Other forms of progressive inherited retinal degeneration include cone degeneration associated with cone cell death, cone–rod dystrophy wherein the cones die first followed by the rods, and Leber congenital amaurosis (LCA), which is linked to a severe loss of vision either at birth or in the first year of life. One of the most common genes associated with LCA is *RPE65* expressed in the retinal pigment epithelium (RPE) cells [11]. Non-progressive retina degeneration includes stationary congenital night blindness (SCNB) [12]. Some mutations in Rho, but also other proteins expressed in rod cells, may cause this form of inherited retina degeneration.

## 2. Genetic and Molecular Mechanisms of Retinal Degeneration

### 2.1. Overview of Genetic Mutations

To date, over 70 genes expressed in the retina have been identified to be associated with RP “https://retnet.org/ (accessed on 30 September 2024)”. The following genes cause RP most frequently: (i) adRP-related genes *RHO* (rhodopsin), *PRPH2* (peripherin 2), *RP1* (retinitis pigmentosa 1), and *PRPF3*1 (pre-mRNA processing factor 31); (ii) arRP-related genes *IMPDH1* (inosine monophosphate dehydrogenase 1), *PRPF8* (pre-mRNA processing factor 8), *USH2A* (usherin), *ABCA4* (ATP binding cassette subfamily A member 4), *PDE6A* (phosphodiesterase 6A), *PDE6B* (phosphodiesterase 6B), *RPE65* (retinol isomerohydrolase RPE65 or retinal pigment epithelium 65), and *EYS* (Eys shut homolog); (iii) X-linked RP-related *RPGR* (retinitis pigmentosa GTPase regulator) and *RP2* (retinitis pigmentosa 2); and (iv) syndromic RP-related genes, including *ABCA4* and *MYO7A* (myosin VIIA). Among them, *RHO* is the most common gene, which accounts for 20–30% of adRP and 10% of all RP cases [10].

### 2.2. Structure of Rho

Rhodopsin (Rho) is a visual receptor belonging to the heptahelical G protein-coupled receptor (GPCR) family, predominantly expressed in the specialized disc membranes of rod photoreceptor outer segments (Figure 1B,C). It is composed of seven transmembrane helices (TM1–TM7), connected by three extracellular loops (ECL1–ECL3) on the intradiscal side and three intracellular loops (ICL1–ICL3) on the cytoplasmic side. Key structural components such as helices TM3, TM6, and TM7, along with ECL2 and ECL3, contribute to the formation of the ligand-binding pocket [13].

ECL2 adopts a twisted two-stranded β-sheet structure that acts as a stabilizing “plug” for Rho’s natural ligand, 11-*cis*-retinal, within the chromophore-binding pocket [14]. Hydrophobic residues within this site further enhance ligand stabilization. The 11-*cis*-retinal chromophore binds to the apoprotein opsin via a protonated Schiff base covalent bond and is essential for photon capture. Upon illumination, 11-*cis*-retinal undergoes isomerization to all-*trans*-retinal, triggering conformational changes in the protein that lead to the formation of the photoactivated state known as Meta II [15,16]. This conformational shift facilitates the binding of the heterotrimeric G protein transducin at the cytoplasmic effector site of Rho, initiating the phototransduction cascade [17,18,19]. Additionally, Rho interacts with other proteins such as Rho kinase and arrestin at this site to terminate signaling and restore its inactive state [20,21].

The N-terminal region of Rho contains two glycosylation sites at Asn2 and Asn15, which are critical for receptor maturation and its transport to the outer segment discs [22,23]. This region also plays an essential role in the proper folding and stabilization of the receptor. The C-terminal tail contains a VXPX motif that is central to correct receptor transport [24]. It also contains two palmitoylation sites at Cys322 and Cys323 [25]. These sites facilitate the anchoring of Rho to the membrane bilayer, which is vital for its functional integrity.

Mutations in structurally and functionally important regions of Rho can impair its stability, folding, transport, or signaling, and are associated with RP. These mutations highlight the critical roles of Rho’s structural elements in maintaining its function and overall visual health.

### 2.3. Classification of Mutations in Rho

Mutations in Rho are categorized into seven distinct classes based on their effects on the receptor’s structure and function: (i) Class 1, mutations altering the receptor transport without causing protein misfolding; (ii) Class 2, mutations that disrupt the correct protein folding, cause ER retention, and affect stability; (iii) Class 3, mutations impairing endocytosis; (iv) Class 4, mutations that alter posttranslational modifications; (v) Class 5, mutations affecting signaling; (vi) Class 6, mutations that result in constitutive receptor activation; and (vii) Class 7, mutations disrupting dimerization of Rho. This class was recently added to the list based on the results reporting disruption of the Rho dimer formation by F45L, V209M, and F220C substitutions [26,27]. However, the follow-up research revealed that these Rho mutants can dimerize within the plasma membrane of cultured cells albeit with a distinct dimerization propensity from the wild-type (WT) receptor [28,29]. Despite this classification, a significant number of Rho mutations remain unclassified. Nevertheless, recent advances in deep mutational scanning approaches suggest that most of the previously uncharacterized mutations likely belong to Class 2 [30]. This highlights the prevalence and pathological significance of defects associated with misfolding and instability in Rho-linked RP. In humans, the most prevalent mutation in the *RHO* gene related to adRP accounts for the substitution of Pro23 to His ([31] and https://www.nei.nih.gov/ accessed on 30 September 2024). Thus, research involving Rho P23H remains a major focus in understanding the underlying mechanisms and pathological consequences of RP.

### 2.4. Structural Basis of Rho Misfolding

Properly folded and functionally stable Rho that can reach the rod outer segment membranes is critical to maintaining a healthy retina [32]. The correct fold of the receptor, its stability, and the binding of its ligand 11-*cis*-retinal, which are critical for receptor integrity and trafficking, depend on the specific local residue interaction networks [32,33,34]. Key amino acids located near the chromophore-binding site, the N-terminus, the ECL2, and the extracellular regions of TM2, TM3, TM5, TM6, and TM7 helices, as well as near the G protein recognition site, are involved in these internal contacts [35,36]. The Cys110 and Cys187 residues that form a critical disulfide bond serve as an anchor for many interactions between the chromophore and the neighboring residues, including E113, Glu122, Trp126, Phe203, His211, Trp265, Tyr268, and Lys296. Asp78, Arg135, Pro171, Tyr178, Pro180, Glu181, Asp190, Met207, His211, and Lys296 are the residues located within the helices that are fundamental for Rho stability and retinal accommodation within its binding site. The N-terminal residues, including Thr4, Glu5, Thr17, Pro23, and Gln28, are essential for the correct folding of Rho [37]. These key amino acids are specifically prone to mutations with pathological consequences. However, RP-linked mutations in Rho are not restricted to the above-listed regions. Disease-causing mutations could occur anywhere within all the TM helices, connecting loops, and the receptor N- and C-termini (Figure 1B,C). Even the substitution of a single amino acid results in a disruption of the internal structural networks that leads to the death of rod photoreceptors followed by the death of cone photoreceptors. Consequently, the detrimental outcomes of disrupted retina integrity present as progressive loss of vision and eventually blindness [26]. Thus, thorough structural studies of the specific Rho mutants are required to enhance our understanding of key structural determinants, which upon disruption trigger protein misfolding and cause RP.

## 3. Cellular Mechanisms of Inherited Retinal Degeneration

Although it is commonly accepted that an inherited mutation is a trigger of photoreceptor cell death in RP, due to the extremely heterogeneous nature of this pathology, the precise mechanism is still not fully understood. In RP, initially, different cellular processes, including endoplasmic reticulum (ER) stress, calcium overload, cellular unfolded protein response (UPR), and abnormal autophagy, are activated to maintain cellular homeostasis. With the failure to overcome the harmful stressor, oxidative stress, inflammation, programmed cell death, namely apoptosis, and apoptosis-independent cell death signaling are activated and lead to photoreceptor demise. In this review, we summarize the cellular mechanisms and cell death pathways contributing to vision loss in RP. In addition, we discuss emerging pharmacological therapeutic strategies that are being developed to combat RP, a currently incurable blinding disease.

### 3.1. Unfolded Protein Response

**Figure 2:**
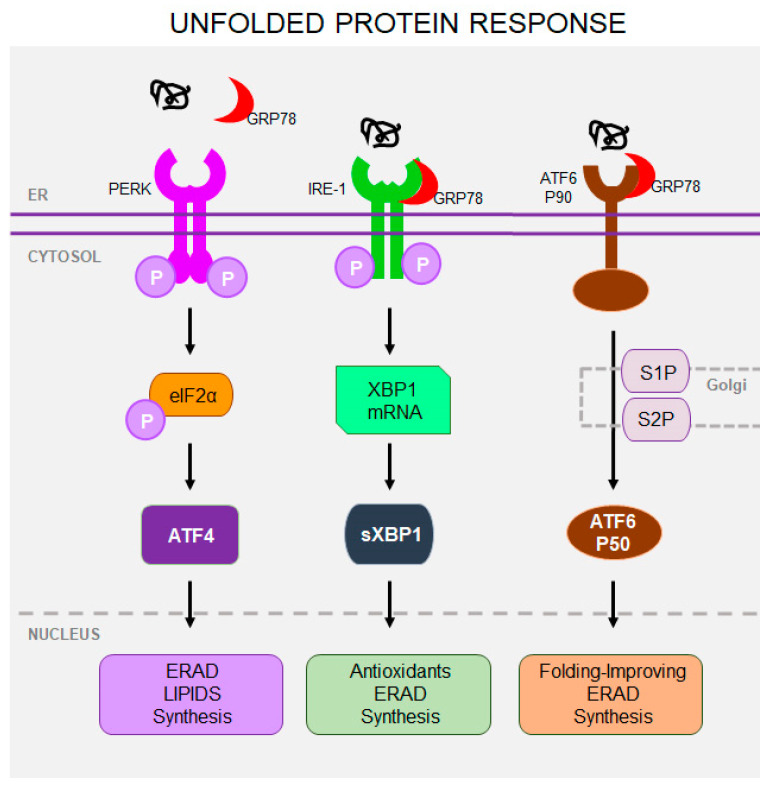
Unfolded protein response. The unfolded protein response (UPR) involves three primary sensor receptors within the ER membranes: protein kinase RNA-like ER kinase (PERK), inositol-requiring enzyme 1 (IRE1), and activating transcription factor 6 (ATF6). PERK phosphorylates eIF2α, which reduces protein translation and upregulates ATF4 transcription factor, which activates the expression of antioxidants and components of the ER-associated degradation ERAD signaling. Activated by unfolded proteins, IRE1 activates transcription factor sXBP1 which stimulates the synthesis of protein folding regulators, ERAD, and lipid biosynthesis. ATF6 (P90), upon activation, translocates to the Golgi apparatus, where it is cleaved to P50 form by proteases S1P and S2P. Cleaved ATF6 stimulates the expression of ERAD and folding regulators.

Misfolding of Rho caused by Class 2 RP-linked mutations results in protein aggregation in the ER [38]. Consequently, protein overload within the ER membranes engages the cellular UPR signaling to reduce the misfolded protein load in order to maintain the viable and functional photoreceptor cells. Three UPR sensors are involved in this regulatory mechanism: protein kinase RNA-like ER kinase (PERK), inositol-requiring protein 1 (IRE1), and activating transcription factor 6 (ATF6) (Figure 2). Failure to adapt to ER stress and restore protein folding homeostasis results in the activation of cell death mechanisms [39,40,41]. Indeed, retention of misfolded Rho in the ER has been demonstrated in several transgenic models of RP, including mice [42], rats [43], Xenopus laevis [44,45], and pigs [46]. In *Rho* P23H *knock-in* mice, most of the mutant protein was degraded through activation of the IRE1 signaling pathway and the ER-associated protein degradation (ERAD) [47,48]. IRE1 directs both autophagy–lysosomal and ubiquitin–proteasomal degradation pathways to remove misfolded Rho [49,50]. Activation of ATF6 is also required for effective clearance of misfolded proteins. Genetic ablation of *Atf6* in the *Rho* P23H *knock-in* mice accelerated retina degeneration in older mice [51]. Prolonged UPR activation stimulates pro-apoptotic PERK signaling to reduce protein synthesis and promote cell death signaling. Inhibition of PERK in transgenic *Rho* P23H-1 rats, a model of the early stage of RP, aggravated ER stress [52]. On the other hand, long-term stimulation of the PERK signaling in *Rho* P23H *knock-in* mice induced the nuclear factor erythroid 2-related factor 2 (NRF2), a transcription factor, which is associated with antioxidant responses [53]. These results suggest that PERK and its downstream signaling pathway initially serve as a protective cellular mechanism. However, all these intrinsic mechanisms designed to have a protective role under homeostatic conditions are not sufficient under chronic conditions of continuous exposure to misfolded protein and lose their ability to prevent photoreceptor cell death. Uncontrolled UPR also leads to increased intracellular Ca^2+^ concentration in the retina, triggering activation of calpain-mediated apoptosis of photoreceptors [54].

In addition, Class 2 misfolding Rho mutants often interfere with the biogenesis and trafficking of WT Rho receptors. This so-called dominant-negative effect results in the co-aggregation of WT protein in the secretory pathway, which enhances the degradation of healthy WT receptors, potentiating the alteration of photoreceptor homeostasis [55,56].

There are two common protein degradation systems: the ubiquitin–proteasome system and the autophagy–lysosome system. The ubiquitin–proteasome system selectively degrades proteins by ubiquitination, a substrate modification with ubiquitin molecules, while autophagy is a system that utilizes lysosomes to degrade damaged cellular constituents [57].

### 3.2. Autophagy

Autophagy is a lysosome-mediated degradation process that functions as an intracellular degradation system of damaged proteins or cellular organelles. In Greek, the word “auto” means “self” and “phagy” means “eating”; thus, autophagy is a “self-eating” process involved in the cell repair mechanism prolonging cell survival [58]. Specifically in photoreceptors, autophagy is an adaptive mechanism to control the levels of Rho and other phototransduction proteins to maintain retinal homeostasis. Although autophagy in the visual system is not fully understood, components of the autophagy pathway were found in most eye structures, including the retina. In the retina, markers of autophagy were detected in the inner nuclear layer (INL), the outer nuclear layer (ONL), the ganglion cell layer (GCL), and the RPE cells. Among these, autophagy-related (ATG) proteins are involved in the formation of autophagosomes that are further fused with lysosomes to degrade autophagosomal load within the autophagolysosome [57]. Indeed, autophagy is critical for the clearance of proteins involved in phototransduction in order to maintain healthy photoreceptors. Depletion of *Atg5* in rod photoreceptors reduced autophagy and increased photoreceptor cell death [59]. Lysosomal degradation is also involved in the clearance of misfolded proteins in photoreceptors and is often dysregulated under persistent stress of inherited misfolding mutations, which enhances photoreceptor demise as demonstrated in *Rho* P23H *knock-in* mice [60].

### 3.3. Proteasomal Degradation

The ubiquitin–proteasome is a common mechanism used for the degradation of unwanted proteins regulating various cellular processes. Proteins that need to be degraded are decorated with ubiquitin by ubiquitin-activating enzyme (E1), ubiquitin-conjugating enzyme (E2), and ubiquitin ligase (E3). Polyubiquitinated proteins are recognized and degraded by the 26S proteasome, a barrel-like structure, containing protein-degrading enzymes in its core [57]. In RP caused by misfolded Rho due to a high load of receptor aggregates needing to be removed, the action of the ubiquitin–proteasome system is often insufficient under this chronic condition. In addition, based on the study in *Rho* P23H *knock-in* mice, abnormal activation of autophagy counters the protective effects initially provided by proteasomal degradation of improperly folded receptors with deleterious consequences of progressive photoreceptor degeneration [50].

## 4. Pathophysiological Pathways

The inability to overcome mutation-induced cellular stress activation, various pathological pathways such as oxidative stress, metabolic stress, inflammation, and immune responses contribute to the retina remodeling, and release of proinflammatory and pro-apoptotic factors that consequently lead to progressive photoreceptor degeneration and ultimately blindness.

### 4.1. Oxidative Stress in RP

The reactive oxygen species (ROS) and nitrogen species (RNS) are produced in response to metabolic demand or external stimuli [61]. ROS/RNS are primarily generated in the mitochondria during cellular respiration and they are required to maintain cellular homeostasis. Under normal physiological conditions, these potentially harmful species are neutralized by mitochondrial and cellular antioxidant defense mechanisms, including specific enzymes that work to maintain oxidative balance [62]. However, under pathological conditions, this balance can be disrupted, leading to excessive accumulation of free radicals. Overproduction of these oxygen-reactive species and their accumulation within the cell leads to modifications of lipids, proteins, and nucleic acids, resulting in a decline in key components of an antioxidant defense system leading to oxidative stress, damaging cellular structures, and impairing cell function [63].

The retina is an energy-demanding tissue, requiring a constant supply of nutrients to sustain efficient signal transduction and metabolite turnover. This intensive metabolic activity drives multiple pathways, making the retina particularly vulnerable to oxidative stress, one of several key factors contributing to the progression of RP. As photoreceptors degenerate, the reduced oxygen consumption in the retina leads to hyperoxia, which in turn elevates the production of harmful free radicals [64,65,66]. This oxidative environment promotes the accumulation of toxic compounds such as malondialdehyde, protein carbonyls, and 8-oxo-7,8-dihydro-2′-deoxyguanosine (8-OHdG) that accelerate the death of rod and cone photoreceptors. Indeed, increased levels of oxidative stress markers, including 8-OHdG and protein carbonyls, have been detected in the vitreous fluid of RP patients, indicating enhanced oxidative damage in the retinal environment under this condition [65]. Similarly, heightened levels of malondialdehyde, 4-hydroxynonenal (HNE), and 8-OHdG have been observed in the retinas of RP-linked animal models [67,68]. Furthermore, oxidative stress impairs the phagocytic activity of RPE cells, which is essential for photoreceptor renewal. In fact, abnormal RPE cells were derived from induced pluripotent stem cells isolated from an RP patient carrying a mutation in pre-mRNA processing factor 6 (PRPF6). These RPE cells showed decreased phagocytosis, improper polarity, and diminished barrier function, underscoring the role of oxidative stress in RP pathology [69].

The antioxidant defense mechanism is a complex system involving multiple key components that protect cells from oxidative stress. Central to this mechanism are glutamate cysteine ligase (GCL), glutathione (GSH), glutathione peroxidase 4 (GPX4), superoxide dismutases (SOD1 and SOD2), catalase, and heme oxygenase 1 (HO-1).

**Figure 3:**
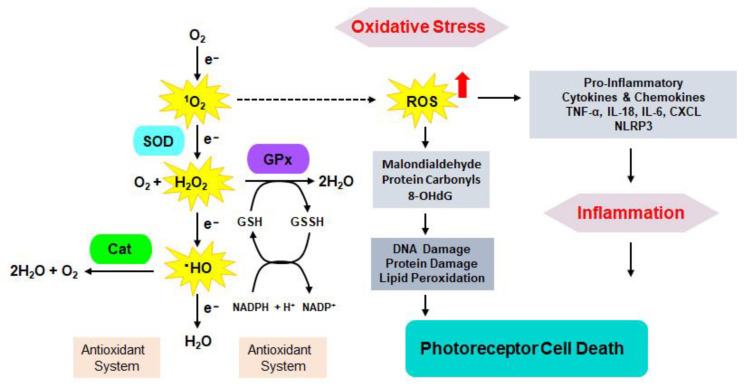
Schematic interplay between oxidative stress, inflammation, and photoreceptor cell death. Oxidative radicals are generated during respiration in mitochondria. Under normal physiological conditions, superoxide dismutase (SOD) catalyzes superoxide radicals (^1^O_2_) into hydrogen peroxide (H_2_O_2_) and oxygen (O_2_), while catalase breaks down hydroxyl radicals ·OH to water (H_2_O) and O_2_. H_2_O_2_ is converted by glutathione peroxidase to H_2_O. During this reaction, GSH is converted to its reduced form GSSH. The back conversion of GSSH → GSH involves NADPH → NAD^+^ change. Excess of reactive oxygen species (ROS) accumulated under chronic conditions of genetic mutation leads to damage of cellular content and release of pro-inflammatory markers that aggravate inflammation, ultimately leading to cell death.

- Glutamate Cysteine Ligase (GCL) is a redox-sensitive homodimer composed of the catalytic subunit containing the substrate binding site, and the regulatory subunit, which modulates the enzyme’s activity. GCL is the primary regulatory enzyme in GSH synthesis.
- GSH is a tripeptide composed of glutamine, glycine, and cysteine that neutralizes reactive oxygen species (ROS). GSH exists in reduced (GSH) and oxidized (GSSG) forms. The elevated ratio of GSSG to GSH signals oxidative stress within the cell [70]. GSH is a co-factor for GPx enzymes.
- GPxs are phospholipid hydroperoxidases that catalyze the synthesis of hydrogen peroxides into water molecules with the conversion of GSH to its oxidized form GSSH (Figure 3). Four isoforms, GPx1-GPx4, are expressed throughout the body. GPx4 plays a crucial role in cell survival by preventing peroxidation of polyunsaturated fatty acids (PUFAs). Retinas are particularly rich in PUFAs and thus prone to oxidation. Impaired GPx4 function is linked to various pathologies, including neurodegenerative diseases [71,72,73]. Genetic ablation of the RPE-specific GPx4 resulted in the acceleration of retinal apoptosis along with a notable loss of photoreceptors [73]. Loss of mitochondrial GPx4 resulted in the accelerated degradation of photoreceptors in the early stage of RP [74]. On the other hand, stimulation of the NRF2/GPx4 signaling delayed the death of photoreceptors in *rd10* mice [68].
- Superoxide dismutases (SODs)**,** specifically SOD1 and SOD2, protect cells by converting superoxide radicals into hydrogen peroxide. This hydrogen peroxide is then further broken down by GPxs and catalase, yet another key enzymes in antioxidant defense, reducing the total ROS levels and thus mitigating oxidative damage (Figure 3). Externally induced upregulation of SOD1 and GPx4 in RPE cells exposed to oxidative stress protected these cells from degeneration [75,76].
- Heme oxygenase 1 (HO-1) is an enzyme that degrades heme into carbon monoxide, iron, and biliverdin, promoting cellular homeostasis. Induced during cellular stress, HO-1 has antioxidant and anti-inflammatory effects, supporting the cell’s adaptive response to oxidative damage [77].

This endogenous antioxidant machinery plays a vital role in maintaining the balance between the generation and degradation of oxygen radicals in a collaborative manner. Due to the rapid degeneration of rod cells in RP, an abnormal surge of oxidants in the retina impairs cones’ redox machinery, which ultimately contributes to overall oxidative stress in RP [78]. However, overexpression of antioxidant enzymes such as catalase, SODs, and GPx4 could counter the generation of excessive oxidants in the retina and delayed cones degeneration in *rd10* mice, a model of RP [76,79]. Although overexpression of SOD2 alone was not effective, its co-expression with catalase improved cone survival in *rd1* and *rd10* mice [76].

To maintain the cellular redox balance, the gene expression nuclear factor erythroid 2-related factor 2 (NRF2) contributes to the regulation of this antioxidant machinery [68,80]. Indeed, overexpression of NRF2 in RPE significantly improved the structural stability of RPE cells and the retinal morphology in the *rd1* mouse model [81]. Also, knockout of the UPR-related factor *Atf4* in *Rho* T17M mutant mice accelerated NRF2 activity, suggesting that improvement in antioxidant status, reducing harmful ROS concentration along with reduced ER stress and UPR response, is critical for delaying disease progression in RP [82].

Together, all these antioxidant enzymes form an intricate system that regulates oxidative stress, thereby preserving cellular integrity and function. However, during RP, malfunction of the endogenous antioxidant machinery accelerates disease progression.

### 4.2. Immune Response and Inflammation in RP

In RP, oxidative stress often induces activation of microglia and inflammatory responses which trigger apoptotic pathways resulting in the death of retinal cells [83]. Inflammation plays a critical role in the progression of neurodegenerative disorders, including retinal degeneration such as RP. Similar to the brain, neuroinflammatory responses in RP involve the activation of microglial cells, which are the primary immune cells in the retina. These cells express various cell surface death receptor sites, including Toll-like receptors (TLRs), interleukin-1 receptor (IL-1R), tumor necrosis factor receptors (TNFRs), and CX3C chemokine receptor 1 (CX3CR1), which recognize a broad range of pathogens [84]. Under physiological conditions, Müller glia, the retina-specific glial cells, and microglia engage in immune surveillance and provide essential metabolic and functional support for neurons [85,86]. However, under chronic stress caused by pathogenic mutations, dying photoreceptors release damage-associated molecular patterns (DAMPs) which are recognized by TLRs and initiate a defense response [87,88]. Yet, excessive release of DAMPs leads to a pathological immune reaction, which disrupts the blood–retina barrier (BRB) and allows for the infiltration of circulating immune cells, which exacerbate retinal degeneration [89]. Indeed, infiltration of microglia into the photoreceptor cell layer was observed in *rd1* mice at postnatal day 14 (P14) and in *rd10* mice at P21, indicating that microglia are activated in the early onset of RP [90,91]. In addition, increased levels of cytokines (IL-1β, IL-6, TNF-α), chemokines (CCL3, CCL5), and markers of glial regulatory pathways were found in *rd10* mice [92]. In both *rd1* and *rd10* mice, uncontrolled secretion of pro-inflammatory mediators such as CCL2 and TNF-α by activated microglia worsened disease progression [93,94].

**Figure 4:**
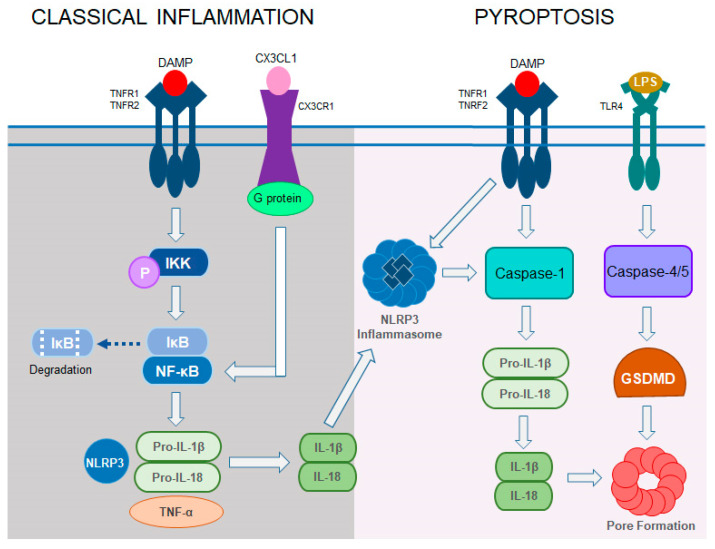
Classical inflammation and pyroptosis signaling. In classical inflammation, damage-associated molecular patterns (DAMPs) activate phosphorylation of IκB kinase (IKK), which degrades IκB from the IκB/NFκB complex leading to the activation of NFκB. Activated NFκB stimulates the expression of proinflammatory cytokines, including IL-1β, IL-18, and TNF-α, as well as the expression of NLRP3, which leads to the formation of inflammasome. In addition, chemokine receptor CX3CR1 activated by CX3CL1 stimulates NFκB through G protein signaling. Pyroptosis is activated by DAMPs through death receptors; for example, tumor necrosis factor receptors (TNFR1 and TNRF2), which stimulate the expression of NOD-like receptor protein 3 (NLRP3) and inflammasome formation that activates caspase-1, which activates IL-1β and IL-18. Alternatively, pyroptosis is activated through Toll-like receptor 4 (TLR4) stimulated by bacterial lipopolysaccharides (LPS). Caspase-4 and -5 are activated in this pathway leading to the activation of gasdermin (GSDMD), which inserts into the membrane forming a pore that allows for the release of pro-inflammatory cytokines activated by caspase-1.

TNF-α, a classical inflammatory mediator acting through two receptors TNFR1 and TNFR2, accelerates the phosphorylation of NF-κB, subsequent maturation of proinflammatory cytokines IL-1β and IL-18, and upregulation of the NOD-like receptor protein 3 (NLRP3) inflammasome, thereby contributing to excessive induction of inflammatory responses ultimately leading to cell death (Figure 4) [95]. Increased levels of TNF-α and NLRP3 expression have been observed in RP models, including the *Rho* Q344X mouse model and *Rho* P23H-1 rat model [96,97]. In addition, in *Rho* P23H-1 rats, the activation of caspase-1 contributes to photoreceptor degeneration. However, in *rd10* mice, elevated pro-inflammatory cytokines activate BAX and caspase-3-mediated cell death [97,98]. Also, emerging evidence coming from LPS-induced systemic inflammation in *Rho* P23H-3 rats, a model of the late-stage RP, showed accelerated photoreceptor demise linking systemic inflammatory processes to the progression of retinal degenerative disorders [99].

Chemokine signaling is another key modulator of neuroinflammation. CX3CL1 exerts its neuroimmune regulatory role through a G-protein-coupled receptor (CX3CR1) and activation of NF-κB (Figure 4) [100]. In the retina, activation of CX3CL1/CX3CR1 signaling modulates the inflammatory response and phagocytic activity of microglia [101]. Genetic ablation of CX3CR1 in *rd10* mice aggravated photoreceptor deterioration due to dysregulated microglia activation [102]. However, increasing CX3CL1/CX3CR1 signaling in these mice retinas via exogenous intravitreal delivery of recombinant CX3CL1 decreased microglial infiltration and thus reduced their phagocytic activity, which improved retina morphology and function [103]. On the other hand, inhibition of CX3CL1/CX3CR1 signaling with an allosteric antagonist (AZD8797) delayed photoreceptor death and prevented reactive gliosis in the RD mouse model established by a single intraperitoneal injection of NaIO3 at a dose of 60 mg/kg [104]. These conflicting results suggest that more studies are required to establish the potential of targeting CX3CL1/CX3CR1 signaling as a therapeutic strategy. Nevertheless, together, these results highlight the importance of managing inflammation and related signaling pathways as part of therapeutic strategies for RP [105].

## 5. Photoreceptor Cell Death Pathways

### 5.1. Apoptosis

**Figure 5:**
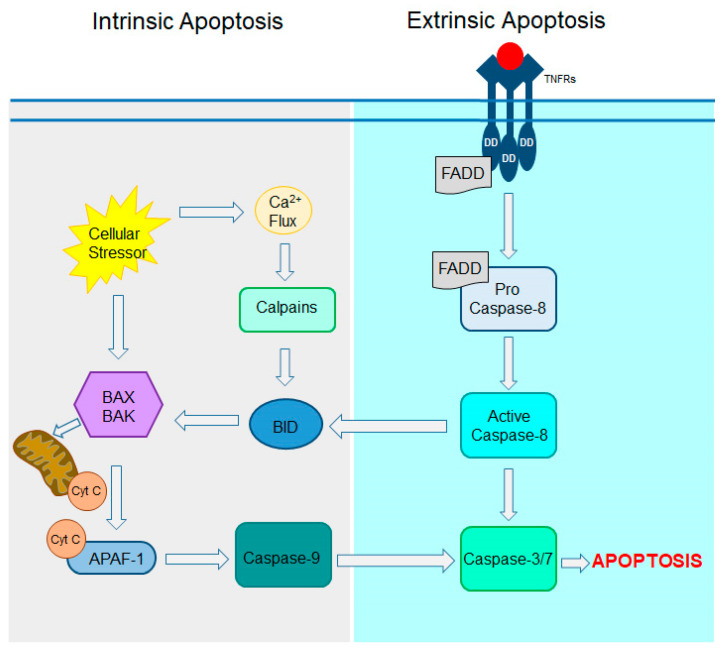
Apoptosis pathway. Extrinsic apoptosis is activated by extrinsic signals through death receptors (TNFRs), which recruit adaptor proteins like the Fas-associated death domain (FADD), followed by pro-caspase-8 activation. Active caspase-8 directly stimulates executioner caspase-3 and -7, leading to apoptosis. Caspase-8 can also stimulate BID, which activates BAX and BAK to permeabilize the mitochondrial membrane, linking the extrinsic and intrinsic pathways. The intrinsic pathway is activated by cellular stressors like damaged DNA or oxidative stress, which activates BAX and BAK. Permeabilized mitochondria release cytochrome c, which binds to apoptotic protease activating factor APAF1 and triggers activation of caspase-9 followed by activation of executioner caspase-3 and -7.

Programmed cell death, namely apoptosis, is a physiological highly regulated process critical for maintaining cellular homeostasis and eliminating damaged or harmful cells. Apoptosis is mediated through two primary pathways: the intrinsic and extrinsic pathways, which lead to the activation of caspases, the proteolytic enzymes that belong to the protease family (Figure 5). Caspases dismantle damaged cells in a controlled manner. An intrinsic pathway involving mitochondria is activated by internal cellular stress, including oxidative stress, damaged DNA, hypoxia, ER stress, or oncogenes. Stress signals activate pro-apoptotic proteins BAX and BAK belonging to the BCL-2 family. Upon activation, these proteins translocate to the mitochondrial membrane where they oligomerize to form a pore allowing for the release of cytochrome c. Cytochrome c binds to apoptotic protease activating factor-1 (APAF-1), which initiates a cascade mechanism activating caspase-9 followed by the activation of executioner caspases (caspase-3 and 7), resulting in cell death [106]. On the other hand, the induction of apoptosis through the extrinsic pathway occurs when external stressors bind to death receptors such as Fas receptor (CD95), TNF receptor-1 (TNFR1), and tumor necrosis factor-related apoptosis-inducing ligand (TRAIL) receptors (TRAILR1 and TRAILR2), belonging to the TNF receptor superfamily. The binding of a ligand leads to receptor trimerization followed by recruitment of the intracellular adaptor protein Fas-associated death domain protein (FADD), which binds to the death domain (DD) of the receptor. The receptor–FADD complex ultimately recruits pro-caspase-8 (or pro-caspase-10 in humans), which binds to the death effector domain forming the death effector signaling complex. This complex activates the executioner caspases (caspase-3 and 7). In some cases, a small amount of active caspase-8 enhances the apoptotic process by activation of the BCL-2 family member BID which stimulates the BAX/BAK-dependent intrinsic apoptotic signals [107,108]. Both intrinsic and extrinsic pathways converge on the activation of executioner caspases. In both scenarios, apoptotic cells display an “eat-me” signal through phosphatidylserine exposed on their cell surface that attracts phagosomes to clear these cells without inducing inflammation.

Activation of different executioner caspases was reported in different RP models. Increased caspase-3 activity was detected in *rd1* and *rd10* mice. However, genetic depletion of caspase-3 in *rd1* mice did not delay photoreceptor degeneration [109]. In RP caused by mutations in *Rho*, including P23H, T17M, and S334ter, activation of ER stress triggers a Ca^2+^ release from the ER membranes, which increases intracellular calcium levels [110,111]. In *Rho* P23H-3 rats, excessive calcium concentrations stimulated cysteine proteases, namely calpains, which activate caspase-12 and caspase-9 [112]. This signaling coincided with the activation of BAX and cytochrome c release through the mitochondrial membrane [111]. Genetic depletion of caspase-12 significantly delayed retina degeneration in *Rho* T17M mice [113]. Ablation of downstream caspase-7 also protected photoreceptors in these mice [114]. Blocking of calpains activity reduced levels of caspase-7 in *Rho* P23H *knock-in* mice and consequently slowed down photoreceptor cell death [53]. In addition, in both *Rho* P23H and *rd10* mice, retina responses to external stressors share the same pathway involving the Fas receptor. Reducing Fas activity had beneficial effects on retina health in both mouse models [115].

Apoptosis is the main cell death pathway activated in RP. However, under this condition, photoreceptors also degenerate through other programmed cell death mechanisms such as necroptosis, ferroptosis, and pyroptosis described below.

### 5.2. Necroptosis

**Figure 6:**
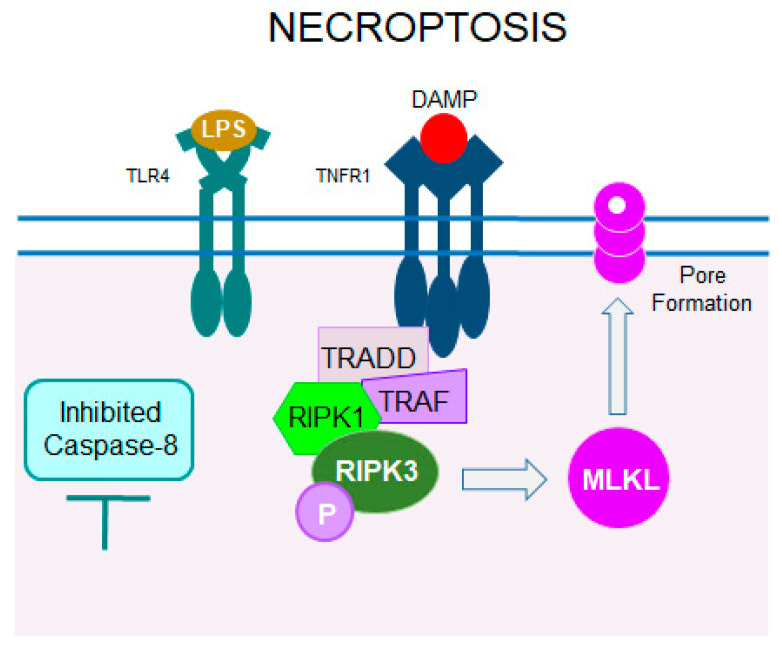
Necroptosis signaling. Necroptosis is triggered by the activation of death receptors, particularly tumor necrosis factor receptor 1 (TNFR1) upon binding of TNF-α. It could also be activated by Toll-receptor 4 (TLR4). TNFR1 recruits adaptor proteins TRADD, TRAF2, and RIPK1. In apoptosis, receptor-interacting protein kinase-1 (RIPK1) is polyubiquitinated and promotes cell survival. When caspase-8 is blocked, RIPK1 interacts with RIPK3, forming a necrosome complex. RIPK3 autophosphorylates and then phosphorylates mixed-lineage kinase domain-like protein (MLKL), a necroptosis key effector, which isomerizes and translocates to the membrane where it forms a pore enabling the release of cellular content. This can further lead to the activation of inflammatory response through released DAMPs.

Necroptosis combines features of regulated apoptosis with uncontrolled cell death necrosis. This caspase-independent mechanism is triggered by the activation of cell death receptors, particularly TNFRs either by cellular or extracellular stressors (Figure 6). Upon receptor activation, the central players of necroptosis, receptor-interacting protein kinase 1 (RIPK1) and RIPK3 form a complex known as the necrosome. Then, RIPK3 activates the final effector of necroptosis called mixed-lineage kinase domain-like protein (MLKL). Activated MLKL oligomerizes and translocates to the plasma membrane where it induces its permeability, enabling the release of cellular content. This can further lead to the activation of inflammatory response through released DAMPs. Necroptosis acts as a backup mechanism when apoptosis is insufficient to ensure the effective removal of damaged cells [116]. In the RP *Rho* P23H-3 rat model, by performing a reverse phase protein array, authors detected the activation of multiple cell death mechanisms with distinct activation of RIPK3 and phospho-MLKL, and suggested necroptosis as the predominant mechanism of photoreceptor death in these rats [117]. In *Rho* P23H-1 rats, necroptotic signaling was also found as a main driver of rod photoreceptor cell death, while NLRP3 inflammasome activation was involved in the secondary cone cell death [97]. On the other hand, in the *Rho* S334ter rats, rod photoreceptor cell degeneration occurred through caspase-mediated signaling, while necroptosis was the main driver of cone degeneration [118]. Elevated expression of RIPK3 was also detected in *rd10* mice in the phase of cone cell death [119].

### 5.3. Ferroptosis

**Figure 7:**
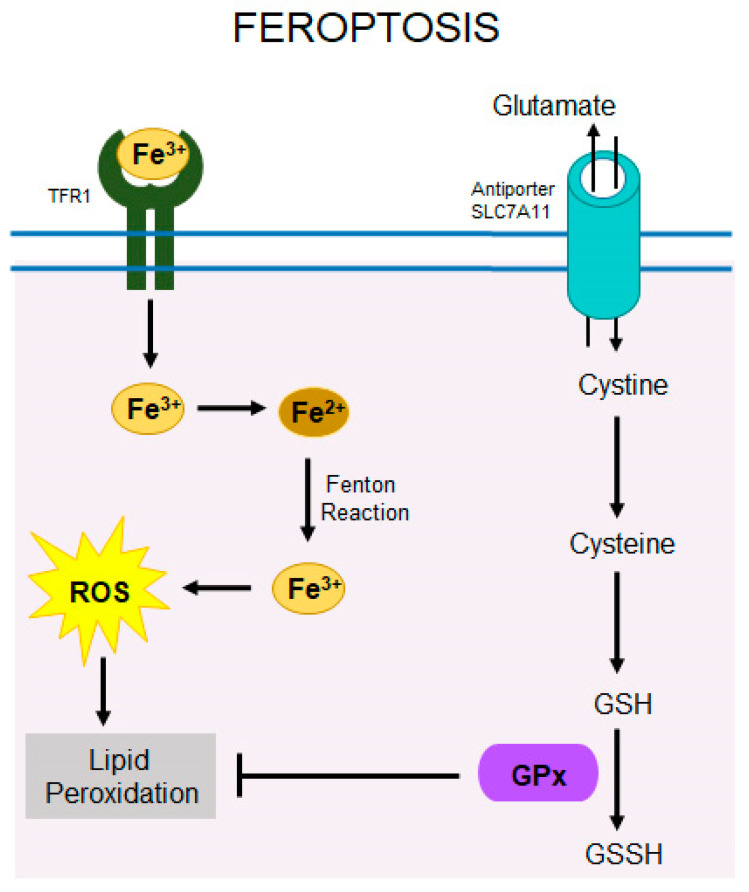
Ferroptosis signaling. Cellular iron is imported via the transferrin receptor (TFR1), which binds Fe^3+^ (ferric iron)-loaded transferrin. Inside the cell, Fe^3+^ became reduced to Fe^2+^ (ferrous iron). Free Fe^2+^ can catalyze the Fenton reaction leading to the generation of reactive oxygen species (ROS) production, which oxidizes unsaturated membrane phospholipids. Under normal physiological conditions, an antioxidant system involving glutathione peroxidase (GPx) prevents lipid peroxidation using its cofactor GSH, which is generated in exchange for glutamate transported out of the cell through the antiporter SLC7A11. Under chronic stress of pathogenic mutations, unchecked lipid peroxidation disrupts membrane integrity and leads to photoreceptor cell death.

Ferroptosis is a recently characterized form of regulated cell death distinct from apoptosis, necrosis, or autophagy (Figure 7). It is driven by the iron-dependent ROS generation through Fenton chemistry that promotes lipid peroxidation leading to oxidative damage of cell membranes [120]. Growing evidence suggests that ferroptosis plays a significant role in neurodegenerative diseases and is one of many cell death pathways contributing to photoreceptor cell death in RP. In support of this, treatment of *rd10* mice with iron chelator deferoxamine decreased levels of ferritin and lipid peroxidation in the retina of these mice [121]. In another study, treatment of *rd10* mice with iron chelators such as VK28 and VAR10303 enhanced the survival of cone photoreceptors and improved visual function [122]. Antioxidant enzymes, including SOD1, GSH, and GPx4, are critical for protecting cells against ferroptosis. Depletion of GSH or inhibition of GPx4 enhances cells’ vulnerability to ferroptosis [116]. In *rd10* mice, deficiency of SOD1 exacerbated oxidative damage of photoreceptors, which could be mitigated by co-expression of SOD1 and GPx4 underscoring their synergistic protective roles. [79].

### 5.4. Pyroptosis

Pyroptosis is a pro-inflammatory-mediated programmed cell death (Figure 4). In photoreceptors, pyroptosis is triggered by DAMPs released from degenerating cells. DAMPs interact with death receptors leading to the expression of NLRP3 and assembly of inflammasome that induces maturation of caspase-1 followed by maturation of IL-1β and IL-18, and cytosolic protein gasdermin D (GSDMD). GSDMD accelerates the secretion of pro-inflammatory cytokines by forming pores in the membrane and subsequent rupture of the cell [123]. Pyroptosis has been implicated in retinal degeneration, particularly in *rd10* and *Rho* P23H mouse models of RP. In these models, increased activation of caspase-1 and the NLRP3 inflammasome, as well as the release of pro-inflammatory cytokines, highlight the role of pyroptosis in photoreceptor cell death [124]. Genetic ablation of caspase-1 in *rd1* mice has been shown to protect photoreceptors, further emphasizing the contribution of pyroptosis to retinal degeneration [125].

Altogether, photoreceptor cell death in hereditary retinal degenerative diseases results from a complex interplay of multiple cell death mechanisms, including apoptosis, necroptosis, ferroptosis, and pyroptosis. Each pathway is driven by distinct molecular triggers and contributes uniquely to the progression of retinal degeneration. A complete understanding of these mechanisms would provide valuable insights into disease pathogenesis critical for the development of therapeutic interventions to preserve vision.

## 6. Pharmacological Treatment Strategies

Proper protein folding, stability, and transport are essential for maintaining the structural and functional integrity of the retina, ensuring its physiological processes and overall health. However, mutations in critical proteins, particularly Rho, can disrupt these processes. Such mutations often result in receptor misfolding, retention in ER, and activation of cell stress signaling pathways, ultimately leading to photoreceptor degeneration. To counteract the pathological effects of mutant proteins, various strategies have been developed. These include (i) modulation of the cellular pathways related to cell stress and endogenous protein quality control, (ii) development of molecules that promote degradation of misfolded proteins to decrease the ER overload, and (iii) development of small-molecule chaperones that improve the folding and trafficking of misfolded proteins. Below, we describe and summarize in Table 1 and Table 2 the progress and potential of these strategies in addressing the challenges posed by misfolding mutant protein-related retinal disorders.

### 6.1. Modulation of the UPR Signaling and Endogenous Protein Quality Control

Accumulation of misfolded proteins induces ER stress and UPR pathways. Under normal physiological conditions, the ER chaperone BiP (GRP78) interacts with nascent proteins to promote their folding and maintain homeostasis [126]. However, under chronic stress caused by mutations, BiP also activates UPR and proteasomal degradation of misfolded proteins to avoid their crowding in the ER lumens. Overexpression of BiP in cultured cells expressing Rho P23H reduced its ER aggregate levels and, in the *Rho* P23H rat model, it reduced death of photoreceptors [43,127]. Thus, enhancing the expression of BiP could be a viable treatment option to prevent pathology related to protein misfolding.

Although activation of UPR signaling in response to ER stress is an adaptive mechanism, prolonged UPR activation can activate apoptotic cell death, suggesting that inhibition of the UPR signaling pathway could prolong cell survival. However, multiple studies involving modulation of the expression levels of the UPR signaling components in different mouse models produced conflicting results, with some indicating the induction of UPR being a protective mechanism, while other studies suggested that it rather accelerates the disease pathology. For example, lowering the expression level of CHOP, which is a downstream effector of PERK signaling in *Rho* T17M mice, was detrimental to photoreceptor survival and function, while in *Rho* P23H transgenic mice it slowed down photoreceptor degeneration [128,129]. On the other hand, in *Rho* P23H *knock-in* mice, genetic ablation of CHOP did not affect the retina health [47]. In addition, inhibition of PERK with the specific inhibitor GSK26066414 enhanced retina degeneration [52]. On the contrary, inhibition of IRE1-dependent RNA cleavage rescued photoreceptors in the *Rho* P23H-1 rat model [130]. Therefore, although the idea of modulation of the UPR pathways is interesting, more studies are needed to elucidate the exact role of each UPR branch in different models of RP to learn whether modulators of the UPR signaling could offer a promising strategy to mitigate RP pathology associated with Rho misfolding.

### 6.2. Modulation of Misfolded Protein Degradation

Lysosome activation is triggered in the retina in response to misfolded Rho as an adaptive survival mechanism to remove improperly folded receptors [131]. Depletion of *Atg5*, one of the autophagosome-specific markers, reduced autophagy and exacerbated rod photoreceptor cell death [59]. On the other hand, administration of an autophagy inducer rapamycin to *Rho* P23H-3 rats slowed the degeneration of rod photoreceptors [111,132]. Rapamycin is an mTOR signaling activator. Upregulation of mTOR signaling by the UPR was found in *Rho* T17M mice and *Rho* P23H-3 rats [111]. Modulation of this pathway showed delayed photoreceptor apoptosis in RP models, highlighting mTOR signaling as a potential therapeutic target. However, autophagy activation is not universally beneficial. Treatment of *Rho* P23H-3 rats with rapamycin failed to prevent cone degeneration, highlighting the complexity of autophagy’s role in retinal health [111,132]. In addition, in *Rho* P23H *knock-in* mice, elevated autophagy flux correlated with worsening retinal degeneration. Pharmacological stimulation of autophagy using CCI-779, a rapamycin analog, exacerbated photoreceptor loss, whereas pharmacological inhibition with hydroxychloroquine or genetic deletion of *Atg5* improved photoreceptor structure and function in these mice [60]. Together these findings suggest that ER stress-induced autophagy, driven by misfolded Rho, can overwhelm the proteasome and inadvertently trigger apoptotic cell death in photoreceptors. Thus, while autophagy plays a protective role under some conditions, its excessive activation may contribute to retinal degeneration. Modulating autophagy, specifically by decreasing its activity in contexts where it exacerbates cell stress, could represent a more effective therapeutic strategy for RP caused by protein misfolding.

Targeting the ubiquitin–proteasome system (UPS) has been proposed as a promising strategy for combating retinal diseases, including inherited blindness such as RP [57]. In RP related to misfolded Rho, chronic stress caused by an accumulation of misfolded receptors in the ER membranes can overwhelm the proteasomal degradation capacity, contributing to cellular dysfunction and degeneration. Thus, enhancing proteasome activity could potentially help alleviate this burden. Indeed, an overload of misfolded Rho P23H could be overcome by overexpression of the 11S proteasome cap subunit, PA28α, which increased ubiquitin-independent protein degradation and delayed photoreceptor degeneration in the mouse model [133]. This finding highlights the therapeutic potential of enhancing proteasomal activity to mitigate the toxic effects of misfolded Rho. However, due to the high complexity of the UPS, the lack of specificity of small molecules targeting proteasomal components and their toxicity pose a challenge to developing safe and effective proteasome modulators to restore proteostasis without unintended side effects.

### 6.3. Small Molecules Targeting Cell Death Pathways

Apoptosis is the common pathway involved in photoreceptor cell death. The mitochondrial-mediated apoptosis requires the activation of BAK and BAX proapoptotic proteins that belong to the BCL-2 superfamily. Upon activation, these proteins oligomerize, forming a pore at the mitochondrial outer membrane that allows for the release of apoptogenic factors, including cytochrome c and apoptosis-inducing factor (AIF) [134,135]. The activation of BAX involves cathepsin D, which is activated by calpains through the degradation of a cytosolic chaperon that sequesters BAX. Upregulation of BAX along with downregulation of its counterpart anti-apoptotic protein BCL-2 has been found in several models of RP, including transgenic *Rho* P23H mice and *rd1* mice [136]. Inhibition of calpains with the calpastatin inhibitor was able to strongly inhibit photoreceptor cell death in *rd1* mice but it only partially slowed degeneration in retinas of *Rho* P23H transgenic mice, probably due to the only partial activation of BAX through the calpain–cathepsin D pathway in RP-associated dominant effect of misfolding mutation in Rho. Inhibition of calpains with calpeptin was beneficial for retina health in another model of RP, *Rho* P23H-1 rats. In addition, photoreceptors in BAX and BAK-deficient mice were protected from pathological death induced by bright light [137]. These results attracted the idea of developing small-molecule BAX inhibitors as neuroprotective agents. As BAX is ubiquitously expressed, such inhibitors could have a wide range of applications. For example, some of these efforts resulted in the development of a series of cell-penetrating BAX inhibiting peptides (BIPs) derived from the BAX binding domain of Ku70 [138]. However, due to the high concentrations required to detect their pharmacological effects, their use as clinical therapeutics is limited. Nevertheless, further efforts recently resulted in the discovery of a potent and orally bioavailable small molecule inhibiting BAX, M109S [139]. Pretreatment with M109S prevented retina damage induced by bright light injury in mice vulnerable to light insult, in a concentration-dependent manner. [139]. M109S also attenuated cell degeneration of ganglion cells induced by optic nerve crush in mice [140]. However, the effectiveness of M109S against RP-related photoreceptor death needs yet to be investigated. Nevertheless, these results strongly suggest that targeting proapoptotic BAX and/or BAK offers a therapeutically attractive strategy to rescue neuronal cells from BAX-induced cell death in neurodegeneration.

Despite apoptosis, necroptosis through activation of the RIP1/RIP3/DRP1 pathway is another cell death mechanism in degenerating photoreceptors in RP. Indeed, up-regulation of the RIP1/RIP3/DRP1 axis was found in *Rho* P23H-1 rats [97]. Interestingly, treatment with RIP1 kinase inhibitor necrostatin-1 substantially improved the structural organization and function of the retina in these rats. Thus, targeting the necroptosis pathway presents a new therapeutic strategy for RP related to Rho carrying the P23H mutation.

### 6.4. Treatments Targeting Inflammatory Responses and Oxidative Stress

Inflammation plays an important role in the onset of photoreceptor degeneration and gradual vision loss in RP. Targeting the inflammatory response occurring outside of the rod photoreceptors could block signals triggering photoreceptor degeneration, and thus slow down degenerative processes within photoreceptors. For example, inhibition of microglia activation with dexamethasone, a synthetic anti-inflammatory steroid, in *rd10* mice resulted in lowering the expression of pro-inflammatory chemokines with consequent preservation of survival of cone photoreceptor and cone-mediated vision [141]. Treatment of these mice with minocycline, a semisynthetic tetracycline derivative, another microglia activation suppressant, also showed beneficial outcomes for retinal health and improved retinal survival of photoreceptor cells in these mice [142].

Treatment with antagonists of the TNF-α receptor such as infliximab and adalimumab also delayed photoreceptor deterioration in *rd10* mice [143].

N-acetylcysteine (NAC), an inhibitor of NLRP3, an intracellular sensor that activates NLRP3 inflammasome, was proven effective in various ocular pathologies, including RP. The underlying mechanism of this chemical is related to an inhibition of the inflammatory cascade and scavenging of hydroxyl radicals. Indeed, NAC administered orally reduced cone photoreceptor death in *rd1* and *rd10* mice [144]. In addition, topical treatment also reduced superoxide formation in the retina, resulting in improved visual function in these mice. A phase I clinical trial) with NAC given orally to patients with RP for 24 weeks revealed good tolerability and an overall good safety profile of this compound [145]. The outcome of this treatment was associated with improvements in visual acuity and macular sensitivity. A large, placebo-controlled clinical trial will determine whether oral treatment with NAC can prolong cone survival and diminish visual disability in patients with RP for a longer time. Nevertheless, its unique antioxidant and anti-inflammatory properties, low toxicity, and oral or topical bioavailability make it attractive as therapeutic in ophthalmic disorders.

A natural compound found in bear bile, namely tauroursodeoxycholic acid (TUDCA), has been used to treat visual pathologies for a long time [146]. This compound possesses neuroprotective effects [147] by reducing inflammatory responses [148,149]. In *Rho* P23H-3 rats, treatment with TUDCA preserved the structure and function of cone and rod photoreceptor cells [150]. This treatment also reduced the number of microglia and prevented their activation [151], along with the reduction in the macrophage population in the retinas of these rats. However, to exert health-beneficial effects, high concentrations of this compound are required. This poses a challenge to use TUDCA as a therapeutic strategy until a biodegradable drug delivery system with slow and sustained release within the eye is developed [152].

Natural dietary compounds such as polyphenols ubiquitously present in fruits and vegetables feature anti-inflammatory, antioxidant, and neuroprotective effects [153,154]. These compounds possess broad biological activity and regulatory effects in multiple cellular pathways [155]. Bioactive polyphenolic compounds were proven beneficial in mitigating degeneration in ocular pathologies, including RP [156]. For example, daily administration of curcumin to *Rho* P23H-1 rats between P30 and P70 improved the retinal structural organization and function. This beneficial effect was associated with decreased levels of the ER stress markers in the treated animals [157]. Safranal, the main component of saffron, administered to *Rho* P23H-3 rats twice a week for four months, enhanced photoreceptor survival in these rats as compared to the vehicle-treated control rats [158]. Flavonoids, such as naringenin and quercetin, slowed down the progression of cone cell death in *rd10* mice [159]. Treatment with quercetin delayed retina degeneration also in *Rho* P23H *knock-in* mice due to its antioxidant and anti-inflammatory properties, and possibly its direct modulatory effect on the stability and folding of mutant Rho [160,161]. In addition, natural compounds, such as quercetin or cumin, can upregulate the NRF2-regulated enzymatic antioxidant pathway to exert their therapeutic effects. Upregulation of NRF2 increased antioxidant enzymes and reduced inflammatory mediators, protecting from degeneration in retinal diseases, including RP [157,162]. Exogenous supply of other antioxidants such as lutein and zeaxanthin, vitamin B6, provitamin A, retinal, retinoic acid, carotenoids, and retinol also improved retinal health in model animals and humans with RP [163,164,165,166].

Recently, we found that inhibition of galanin receptor 3 (GALR3) with its specific antagonist SNAP-37899 has beneficial effects against acute retina damage in mice susceptible to bright light injury [167]. GALR3-mediated signaling plays a central role in the pathogenesis of various diseases, including neurodegenerative diseases, and is involved in the regulation of inflammatory responses occurring in these pathologies. Upregulation of GALR3 was found in the retinas of mice injured with bright light, while pharmacological inhibition with a blood–brain permeable antagonist SNAP-37899 or genetic depletion of this receptor halted the light-induced disintegration of photoreceptors in these mice. Activation of the inflammatory stress signaling is a hallmark of RP; thus, the inhibition of GALR3 could be beneficial in this pathology. Indeed, in *Rho* P23H *knock-in* mice featuring mutation-caused chronic retina degeneration, treatment with GALR3 antagonist or genetic ablation of this receptor prolonged the survival of photoreceptor cells, suggesting that targeting GALR3 signaling could be explored as a potential avenue for development treatments for RP.

Together, targeting inflammation in RP proved to help in slowing down the progression of this retinopathy. Thus, it appears to be a promising avenue for the development of a therapeutic strategy for fighting blindness in RP.

**Table 1:** Modulation of cellular pathways as a treatment strategy for RP.

**Modulated Pathway**	**Compound and/or Target**	Effect	In Vivo Model	References
Unfolded protein response (UPR) signaling	Overexpression of BiP Lowering expression of CHOP Inhibition of IRE-dependent RNA cleavage GSK26066414–PERK inhibitor	Positive Detrimental No effect Positive Detrimental	*Rho* P23H rats *Rho* T17M mice *Rho* P23H *knock-in* mice *Rho* P23H transgenic mice *Rho* P23H-1 rats *Rho* P23H *knock-in* mice	[43,126] [127] [128] [47] [129] [52]
Autophagy	Hydroxychloroquine –autophagy inhibitor Genetic deletion of *Atg5* Rapamycin–autophagy inducer Rapamycin analog, CCI-779	Positive Positive Positive Detrimental	*Rho* P23H *knock-in* mice *Rho* P23H *knock-in* mice *Rho* P23H-3 rats *Rho* P23H *knock-in* mice	[60] [60] [110] [60]
Ubiquitin–proteasome system (UPS)	Enhancing proteasome activity by overexpression of the 11S proteasome cap subunit, PA28α	Positive	*Rho* P23H *knock-in* mice	[132]
Apoptosis	Calpastatin–calpains inhibitor Calpeptin–calpains inhibitor Genetic depletion of *Bax/Bak*	Positive Positive Positive	*Rho* P23H transgenic mice *Rho* P23H-1 rats *Rho* P23H-1 rats	[135] [136] [136]
Necroptosis	Necrostatin-1–RIP1 kinase inhibitor	Positive	*Rho* P23H-1 rats	[97]
Inflammation and oxidative stress	Dexamethasone–microglia activation suppressant Minocycline–microglia activation suppressant Infliximab and Adalimumab–TNF-α receptor antagonist N-acetylcysteine (NAC)–NLRP3 inhibitor Tauroursodeoxycholic acid (TUDCA) Curcumin Safranal Quercetin and Narigenin Quercetin Nutraceuticals, including lutein and zeaxanthin, vitamin B6 provitamin A, retinal, retinoic acid, carotenoids, and retinol SNAP-37899–antagonist of Galanin 3 Receptor (GALR3) Genetic depletion of *Galr3* Genetic depletion of *Cx3cr1* AZD8797–CX3CR1 allosteric antagonist Increasing levels of CX3CL1 elevating CX3CR1 signaling	Positive Positive Positive Positive Positive Positive Positive Positive Positive Positive Positive Detrimental Positive Positive	*rd10* mice *rd10* mice *rd10* mice *rd1 and rd10* mice Patience with RP *Rho* P23H-3 rats *Rho* P23H-1 rats *Rho* P23H-3 rats *rd10* mice *Rho* P23H *knock-in* mice RP Patience *Rho* P23H *knock-in* mice *Rho* P23H *knock-in* mice *rd10* mice RD mice generated by NaIO3 i.p. injection *rd10* mice	[140] [141] [142] [143] [145,149,150] [156] [157] [158] [159] [162,163,164,165] [166] [166] [102] [104] [103]

### 6.5. Non-Selective Chemical Chaperones

Molecular chaperones facilitate proper protein folding and enable its maturation and trafficking to their functional place. Several endogenous chaperones are involved in the biogenesis of Rho. Thus, exogenous molecules with pharmacochaperone properties could help to restore the proper folding of misfolded Rho. Non-selective compounds targeting protein misfolding, such as the chemical chaperone 4-phenylbutyrate (4-PBA), have shown potential in mitigating the effects of misfolded Rho. Clinically approved for conditions like urea cycle disorder, sickle cell disease, and cystic fibrosis, a low molecular weight fatty acid 4-PBA has demonstrated efficacy in decreasing aggregation of the Rho P23H mutant in vitro and reducing UPR signaling in mice carrying the *Rho* T17M mutation by promoting protein degradation [55,168]. Despite these encouraging results, 4-PBA failed to improve retinal health in the transgenic *Rho* P23H-1 rat model, even when administered at high concentrations (500 mg/kg) [26]. This outcome indicates the limitations of non-selective approaches in treating retinal degenerative diseases, likely due to insufficient specificity and potential off-target effects. Interestingly, 4-PBA showed promise in addressing the mislocalization of cone opsin in R91W *Rpe65* mice, a model characterized by reduced levels of 11-*cis*-retinal [169]. These findings suggest that, although non-selective chemical chaperones can provide some benefits, target-specific pharmacochaperones are likely to offer greater efficacy and reduced side effects, making them a more promising avenue for therapeutic development in retinal degenerative disorders.

### 6.6. Pharmacochaperones Targeting Rho

The ligand-binding pocket in Rho has a theoretical solvent-accessible surface of 495 Å and it is surrounded mainly by the hydrophobic residues that are involved in the stabilization of the retinal chromophore [170]. This binding site in Rho is large and flexible enough to accommodate ligands different from the natural chromophore 11-*cis*-retinal. The 9-*cis*-retinal analog can bind to apoprotein rod opsin via the protonated Schiff base without affecting the receptor function. These retinoids have the potential to improve the folding and cell surface targeting of the Rho P23H mutant in vitro, protecting cells from the toxic effect of misfolded protein aggregation within the ER membranes [171,172]. This observation initiated an interest in developing pharmacochaperone therapy to rescue the RP pathology. Importantly, currently, treatment with vitamin A, the precursor of 11-*cis*-retinal with limited exposure to light, is the only recommended treatment option for patients with RP related to Rho misfolding [173]. However, this therapy has various outcomes for different Rho mutants with either beneficial or detrimental effects [174,175,176,177]. Thus, the use of retinoids as pharmacochaperones is limited due to differences in their binding effectiveness to different mutants, instability under light conditions, and toxicity of their photoproducts. Increasing the concentration of retinoids in the eye could also result in unnatural levels of all-*trans*-retinal and its byproducts that could aggravate retina degeneration [178]. In fact, more recent randomized clinical trials do not support the beneficial effects of vitamin A therapy in all types of RP [164]. To avoid overloads of the photo byproducts, the potential of non-isomerasable retinal analogs such as the locked 11-*cis*-7-ring-retinal was evaluated [171,179]. As shown, this retinoid improved the folding and trafficking of the Rho P23H mutant expressed in cells without danger of all-*trans*-retinal accumulation in light conditions. These results suggest that specific light-insensitive retinoid analogs could serve as folding correctors potentially without secondary toxicity effects. The positive effects of 11-*cis*-6-ring-retinal were shown in mice vulnerable to bright light-induced retina degeneration [180]. However, in vivo, studies examining the effectiveness of locked retinals in the RP models are still needed.

To overcome potential side effects associated with the use of retinoid-based chaperones, efforts have been directed to develop non-retinoid small-molecule compounds that could bind to Rho mutants, shifting their conformation towards WT-like. These studies resulted in the discovery of several new synthetic compounds, including SRD005825, NSC45012, 13-*cis*-5,8-ERA, YC-001, and RS1 [33,181,182,183,184]. Other studies performed in our laboratory found the pharmacochaperone potential of natural compounds such as flavonoids and chromenone compounds [160,185].

SRD005825 or SHP630 was derived from 9-*cis*-retinal but has no aldehyde group to form a Shiff base bond with opsin and cannot serve as a precursor for retinoic acid. This compound competed with 9-*cis*-retinal for binding to rod opsin, improved maturation and membrane trafficking of Rho T17M mutant, and preserved retina structure and function upon oral administration [184].

A retinoid derivative such as 8-epoxy-13-*cis*-retinoic acid (13-*cis*-5,8-ERA) that has a three-dimensional chemical conformation similar to 11-*cis*-retinal discovered through the virtual screening could improve the mobility of several RP Rho mutants expressed in cultured cells, likely due to its ability to reversibly accommodate within the chromophore binding site without forming a Shiff base bond [33].

NSC45012 (1-(3,5-dimethyl-1H-pyrazol-4-yl)ethanone) contains a five-membered ring, ketone group, and features three methyl groups that are important for its accommodation within the chromophore binding pocket. It competes with 11-*cis*-retinal for binding to unliganded opsin but it has lower affinity than retinal. In the cells expressing the Rho P23H mutant, it enhanced mutant transport from the ER to the plasma membrane, but its effectiveness in the mouse model of RP was not tested [183].

YC-001 (4-(5-chlorothiophen-2-yl)-3-(thiophene-2-yl)-2,5-dihydrofuran-2-on), found through phenotypic cell-based high-throughput screening, was able to rescue the trafficking and maturation of the Rho P23H mutant in mammalian cells [181]. Similarly to 13-*cis*-5,8-ERA and NSC45012, this compound competes with 11-*cis*-retinal for binding to Rho without forming a Schiff base. However, due to the fast elimination, YC-001 had only a minor impact on improving the photoreceptor survival in vivo [186].

RS1 (3-(benzylsulfamoyl)-4-bromo-n-(4-bromophenyl) benzamide) was identified from the virtual screening of Roche’s non-retinoid library compound collection and validated in thermal stability assays [182]. Although RS1 was not membrane permeable, its derivatives RS2–4 improved the cell surface expression of Rho P23H in vitro. The co-crystallization of ligand-free human rod opsin with RS1 confirmed the receptor–ligand interactions within the chromophore-binding site, providing information for further structure-based compound modifications to develop a more effective chaperone compound.

Based on our and other research group studies, natural polyphenolic compounds, such as quercetin and myricetin, can also bind to ligand-free opsin, improving its stability and enhancing pigment regeneration [161,187]. These flavonoids increased cell surface expression of P23H rod opsin in cultured cells stably expressing this mutant. Moreover, treatment with quercetin and myricetin showed beneficial effects for retina health in *Rho* P23H *knock-in* mice, which was associated with the reduction in the ER and oxidative stress markers [160]. A more detailed discussion on the beneficial effects of polyphenolic compounds on visual health can be found in [105,156].

In the follow-up study, from the library of compounds with a natural product scaffold, we identified in silico a compound with a chromenone motif (CR5) [185]. The binding of this compound was validated first in in vitro experiments followed by studies in vivo in *Rho* P23H *knock-in* mice. CR5 partially improved membrane targeting of multiple RP-related Rho variants in cell culture. Interestingly, *Rho* P23H mice treated with CR5 showed enhanced photoreceptor survival evidenced by improved retina morphology and function.

Our recent advances through rod opsin targeted in silico screening identified two novel non-retinoid bioactive small-molecule compounds, JC3 and JC4, with pharmacochaperone properties. These compounds exhibit favorable characteristics, including permeability across cell membranes and the blood–brain barrier (BBB). JC compounds demonstrated high binding affinity to ligand-free rod opsin, with K_d_ of 98 nM for JC4 and 175 nM for JC3. Importantly, JC3 and JC4 improved the membrane trafficking of 36 out of 123 clinically relevant Rho mutants, highlighting their potential to rescue multiple variants of misfolded Rho. In vivo, treatment with these compounds prevented retinal degeneration in mice exposed to bright light insult, and importantly in *Rho* P23H *knock-in* mice. Treatment with JC3 and JC4 enhanced retinal structural organization and increased the intensity of visual responses compared to vehicle-treated controls. Safety evaluations revealed no apparent adverse effects on overall body weight, retinal morphology, or function in WT mice treated with these compounds, supporting their favorable safety profile. These findings suggest that JC3 and JC4 could be developed into clinically effective drugs to delay degenerative processes in RP [188].

Altogether, accumulating evidence demonstrates that Rho is a valid target for the structure-based development of pharmacochaperones to correct the folding in misfolding mutants and rescue the RP phenotype.

**Table 2:** Pharmacochaperones targeting protein folding.

Compound Name	Target	Effect	In Vitro/In Vivo Models	References
4-PBA	Non-selective	Correct Rho trafficking Correct Rho trafficking Correct cone opsin mislocalization	*Rho* T17M mice *Rho* P23H-1 rats *Rpe65* R91W	[167] [26] [168]
11-*cis*-retinal	Rho P23H	Correct Rho trafficking	Cell culture	[171]
9-*cis*-retinal	Rho P23H	Correct Rho trafficking	Cell culture	[171]
11-*cis*-7-ring-retinal	Rho P23H	Correct Rho trafficking	Cell culture	[170,178]
Vitamin A	Various RP types	Slow down RP progression	Patients with RP	[172]
SRD005825 or SHP630	Rho T17M	Correct Rho trafficking Slow down RP progression	Cell culture *Rho* T17M mice	[183]
13-*cis*-5,8-ERA	Rho T17M Rho P23H Rho E181K	Correct Rho trafficking Correct Rho trafficking Correct Rho trafficking	Cell culture Cell culture Cell culture	[33] [33] [33]
NSC45012	Rho P23H	Correct Rho trafficking	Cell culture	[182]
YC-001	A total of 6 Rho mutants, including P23H	Correct Rho trafficking	Cell culture	[180]
RS1 RS2–4 (RS1 analogs)	Rho P23H Rho P23H	Improve protein stability Correct Rho trafficking	Thermal stability assay Cell culture	[181]
CR5	A total of 30 Rho mutants, including P23H	Correct Rho trafficking Slow down RP progression	Cell culture *Rho* P23H *knock-in* mice	[184] [184]
Quercetin	Rho P23H	Correct Rho trafficking Slow down RP progression	Cell culture *Rho* P23H *knock-in* mice	[160,186] [159]
Myricetin	Rho P23H	Correct Rho trafficking Slow down RP progression	Cell culture *Rho* P23H *knock-in* mice	[160] [159]
JC3	A total of 32 Rho mutants, including P23H	Correct Rho trafficking Slow down RP progression	Cell culture *Rho* P23H *knock-in* mice	[187] [187]
JC4	A total of 26 Rho mutants, including P23H	Correct Rho trafficking Slow down RP progression	Cell culture *Rho* P23H *knock-in* mice	[187] [187]

## 7. Other Treatment Strategies

In addition to small-molecule drug development strategies, several other innovative approaches are being actively pursued to provide therapeutic solutions for patients with RP. These include gene therapy, which seeks to correct or replace defective genes; cell-based therapies, which aim to replace degenerated photoreceptor cells; neuroprotective strategies that focus on preserving retinal cell function and delaying disease progression; CRISPR and other gene-editing techniques to precisely repair genetic mutations; and the development of advanced retinal prosthetics to restore vision in cases of severe photoreceptor loss.

This review acknowledges the significance of these alternative therapeutic strategies, as they share a common ultimate goal: to develop effective interventions that prevent or reverse vision loss, thereby improving patients’ quality of life. The progress made in these areas holds tremendous promise and has been extensively documented in recent literature and summarized in multiple review articles [8,9,189,190]. These comprehensive reviews have delved into the advances, challenges, and future directions for each of these approaches, including their clinical trial outcomes, technological innovations, and translational potential. Thus, this article instead focused on small-molecule pharmacochaperones as a distinct and promising avenue for RP treatment.

## 8. Conclusions

Photoreceptor cell death in RP is primarily driven by the chronic insult caused by inherited mutations in the *RHO* gene or other key photoreceptor proteins. These mutations continuously disrupt cellular homeostasis, activating multiple compensatory pathways aimed at preserving retinal cell balance. However, in RP, these adaptive mechanisms ultimately fail, leading to the activation of diverse cell death pathways. One major contributing factor is the overload of improperly folded proteins in the ER, coupled with their insufficient clearance, which triggers apoptosis. As the retina degenerates, reduced oxygen demand further exacerbates oxidative stress, promoting lipid peroxidation and the activation of ferroptosis. Dying rod photoreceptors also activate microglia, leading to neuroinflammation and pyroptosis. In parallel, necroptosis has been implicated in the death of cone photoreceptors.

These findings emphasize the complexity of RP pathology, as multiple cell death pathways converge to drive photoreceptor degeneration. However, the intricate interplay between these mechanisms remains incompletely understood, highlighting the need for further investigation. While efforts to develop therapeutic strategies have focused on targeting mutant receptors and components of cell death pathways, a more comprehensive understanding of these processes and their regulation is critical for creating effective treatments capable of rescuing photoreceptors and preserving vision in RP, regardless of the underlying genetic cause.

## Data Availability

Not applicable.
